# Facemasks and ferrous metallurgy: improving gasification reactivity of low-volatile coals using waste COVID-19 facemasks for ironmaking application

**DOI:** 10.1038/s41598-022-06691-w

**Published:** 2022-02-17

**Authors:** Daniel J. C. Stewart, Lucy V. Fisher, Michael E. A. Warwick, David Thomson, Andrew R. Barron

**Affiliations:** 1 Energy Safety Research Institute, Swansea University Bay Campus, Swansea, SA1 8EN UK; 2 Tata Steel Strip Products UK, Port Talbot, SA13 2NG UK; 3 Arizona Institutes for Resilience (AIR), University of Arizona, Tucson, AZ 85721 USA; 4 Department of Chemistry and Department of Materials Science and Nanoengineering, Rice University, Houston, TX 77005 USA; 5 Faculty of Engineering, Universiti Teknologi Brunei, Bandar Seri Begawan, Brunei

**Keywords:** Chemical engineering, Coal

## Abstract

The global pandemic response to COVID-19 has led to the generation of huge volumes of unrecyclable plastic waste from single use disposable face coverings. Rotary hearth furnaces can be used to recover Zn and Fe from non-recyclable steelmaking by-product dusts, and waste plastic material such as facemasks could be utilized as a supplementary reductant for the rotary hearth furnace (RHF), but their fibrous form makes milling and processing to appropriate sizing for RHF application extremely challenging. A scalable method of grinding facemasks to powder by melting and mixing with Welsh coal dust reported herein provides a solution to both environmental challenges. The melt-blended PPE/coal dust shows a dramatically improved CO_2_ gasification reactivity (E_a_ = 133–159 kJmol^−1^) when compared to the untreated coal (E_a_ = 183–246 kJmol^−1^), because of improved pore development in the coal during the pyrolysis stage of heating and the catalytic activity of the CaO based ash present in the facemask plastic. The results are promising for the application of waste facemasks in recycling steelmaking by-product dusts in rotary hearth furnaces and may also be suitable for direct injection to the blast furnace subject to further study.

## Introduction

As part of the response to the global COVID-19 pandemic caused by the coronavirus SARS-CoV-2 and its variants, many governments and the World Health Organization recommended or required the use of face coverings to suppress transmission of the virus. Although often politically controversial, the benefit of face masks in mitigating the spread of COVID-19 has led to wide scale adoption^1,2^. Although the UK government specifically recommended non-medical grade^3^, reusable fabric face masks many members of the public opted for single use plastic medical-style facemasks. The North London Waste Authority projected UK citizens were discarding 102 million masks a week during the height of the pandemic^4^. At an average mass of 3 g per mask this approximates to a staggering 300 tonnes per week of non-recyclable plastic waste being generated in the UK alone. On a greater scale, during state-wide lockdowns in China it was estimated the Chinese public were using 900 million facemasks per day^5^, which despite the claim of being centrally disposed of, the plastic content cannot be recycled. Much of the global plastic facemask waste is expected to end up in rivers and oceans in the form of microplastic pollution with disastrous effects on aquatic ecosystems^6–9^. At present, the vast majority of this unrecyclable plastic facemask waste is landfilled; however, alternative waste management strategies include gasification to syngas (CO and H_2_) and pyrolysis for direct energy recovery^10,11^. The recyclability of facemasks is complicated by their blended composition, and also the presence of iron wire designed to improve the seal against the wearers nose. Given the scope of the environmental impact, what is needed is a process of recycling or repurposing facemasks as well as related PPE (disposable gowns, hospital bed pads, and privacy curtains), if the process can use essentially whole items without pre-treatment.

The use of plastic waste in ironmaking and steelmaking to reduce demand for extraction of fossil fuel reductants like coal is a promising area of study and application; however, to-date the two key areas where plastics have seen application at commercial scale for steelmaking have been the inclusion of a small component of waste plastic in the coke oven process^12^ and injection at the tuyere of the BF^13^. The rotary hearth furnace (RHF) is an emerging technology for the separation of volatile metals (Zn, Pb etc.) from steelmaking by-product dusts that are too high in volatile metals for direct recycling into the ironmaking process^14–16^. Self-reducing agglomerates prepared from ferrous by-product dusts and a carbon source (coal, coke breeze, blast furnace dust) are charged into a rotating turntable furnace and heated to 1200–1300 °C for a period between 10 and 30 min. The metal oxides in the agglomerate such as Fe and Zn are reduced via carbothermic reduction to yield pellets of direct reduced iron (DRI) and a separated recyclable secondary oxide dust containing the volatile metal components^17^. Previous studies have indicated the CO_2_ gasification reaction is the rate determining step in the reduction process of cold bonded self-reducing iron-carbon agglomerates^18^ although with extremely high reactivity carbon sources heat transfer effects become more significant^19^. An increased gasification reactivity of the carbon source in self-reducing agglomerates for the RHF can therefore lead to improved reduction rates and therefore improved productivity.

Studies into the inclusion of waste plastics into self-reducing agglomerates as a feedstock for RHFs have yielded promising results, showing good reactivity and reduced CO_2_ emission as a result of the hydrogen content of the plastic^20^. Unfortunately, a key issue in the inclusion of waste plastic in self-reducing agglomerates is particle sizing, where fine particle sizes (~ 115 µm) are required to form a composite pellet with adequate strength for processing^21^. Milling of fibrous plastic materials such as facemasks to appropriate sizing is extremely challenging due to their ductility and relatively low softening temperature. Furthermore, the low density of conventionally milled plastic waste makes storage and transport challenging.

Herein we report a new scalable process in which entire plastic facemasks are blended with a Welsh ground coal injection (GCI) coal and the subsequent change in the CO_2_ gasification reactivity of the material is explored. Gasification reactivity of the material was measured using thermogravimetric techniques, as this allowed for direct observation of the reaction without simultaneous metal reduction reactions occurring. If the gasification reactivity of low volatile content coal can be substantially improved by pre-treatment with waste facemask plastic, it may be applicable as a means of economically increasing the productivity of RHF plants through reduced pellet hold times.

## Results

**Figure 1:**
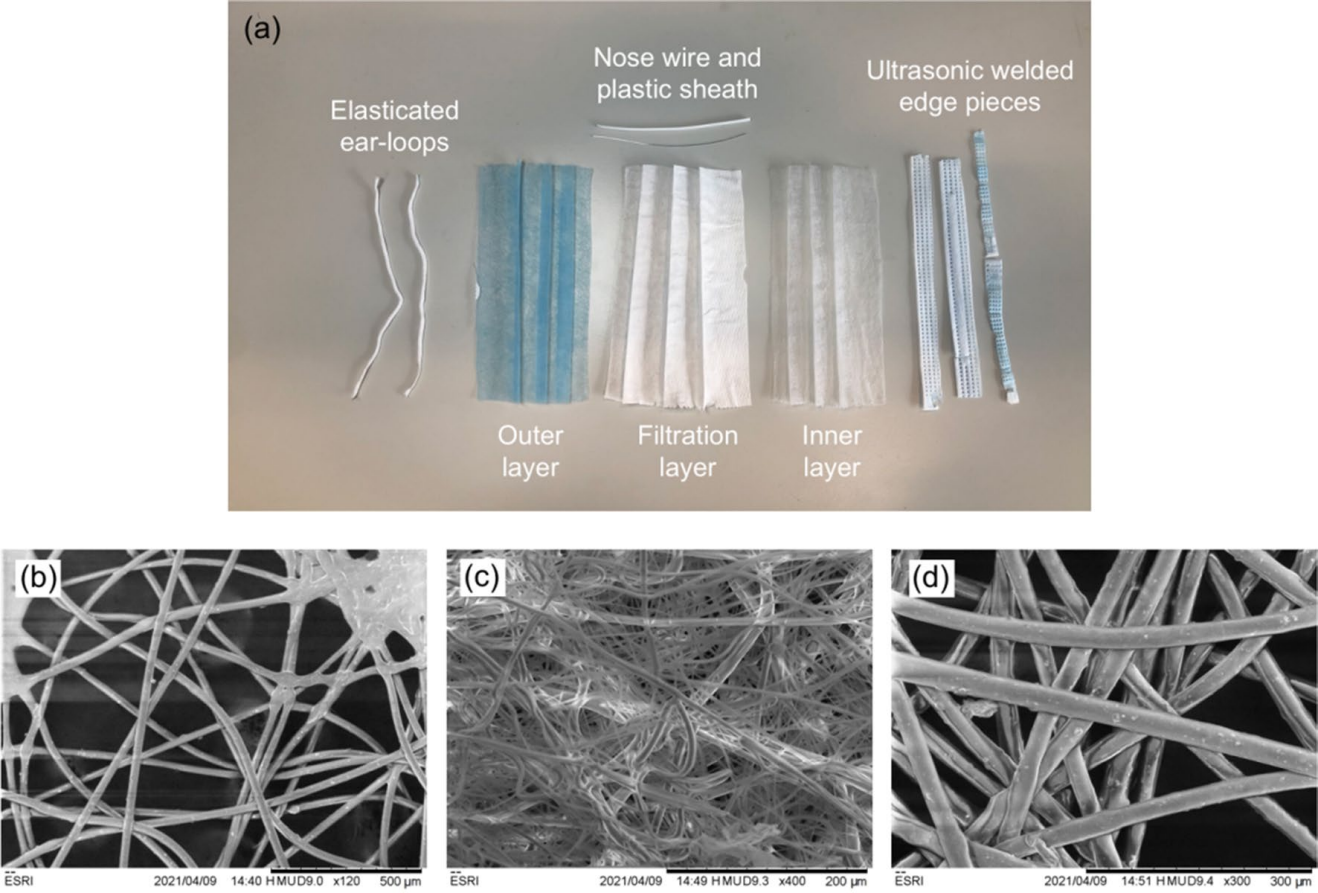
Components of the disposable mask. (**a**) A photograph of a fully disassembled mask with the component pieces labelled. (**b**) SEM image of the untreated mask outer layer. (**c**) SEM image of the untreated mask filtration layer. (**d**) SEM image of the untreated mask inner layer.

Disposable, non-medical grade, facemasks (Changzhou Huangshi Packaging Printing Co. Ltd.) were coarsely chopped into squares using scissors without any prior disassembly. The manufacturer specifies the non-metallic composition of the masks is non-woven plastic (60 wt.%), meltblown plastic (30 wt.%), spandex (7 wt.%) and polyolefin resin (3 wt.%). The masks also contained a small steel wire (ca. 6 wt.% of the total mask) designed to improve the seal against the wearers nose, this wire was not removed during initial processing to better replicate an upscaled process where that would not be feasible. A photographic image of the components is shown in Fig. 1a, while scanning electron microscopy (SEM) images of the layers allowed for better visualization of their fibrous nature. The dense interlocking fibres of the filtration layer can be observed in Fig. 1c, compared with the comparatively less tightly woven fibres in the external (hydrophobic splash resistant) and internal (comfort) layers of the mask in Fig. 1b,d, respectively.

Single use facemasks such as the ones used in this study are typically made of polypropylene fabric. The FTIR spectra of untreated facemask (Figure S1) is in reasonable agreement with Aragaw’s work^22^ characterizing facemask plastic, with the exception that a broad absorbance that was ascribed to cellulose O–H bonding is not observed in the masks used in this study. It appears that the masks used in this work contained no paper component, which given the tremendous variation in manufacturing processes for face coverings in the COVID-19 era and lack of widespread standardization seems likely.

Pulverization of the masks, even by cryogenic ball milling, was found to be totally ineffective. In a similar manner, physically mixing the cut pieces of mask with coal (or charcoal), as per the approach of Wang et al.^23^, did not create a homogeneous mixture. Dankwah et al.^24^ have previously reported that by melting it was possible to physically pelletize iron oxide with a plastic powder. This was done by pulverizing plastic after it had been separately heated to 300 °C and embrittled via quenching. This suggested that the correct physical form for the plastic could be obtained through melt processing. However, in order to obtain a granular material, suitable for producing cold bonded briquettes melt processing was performed by mixing the waste facemasks with coal fines.

**Figure 2:**
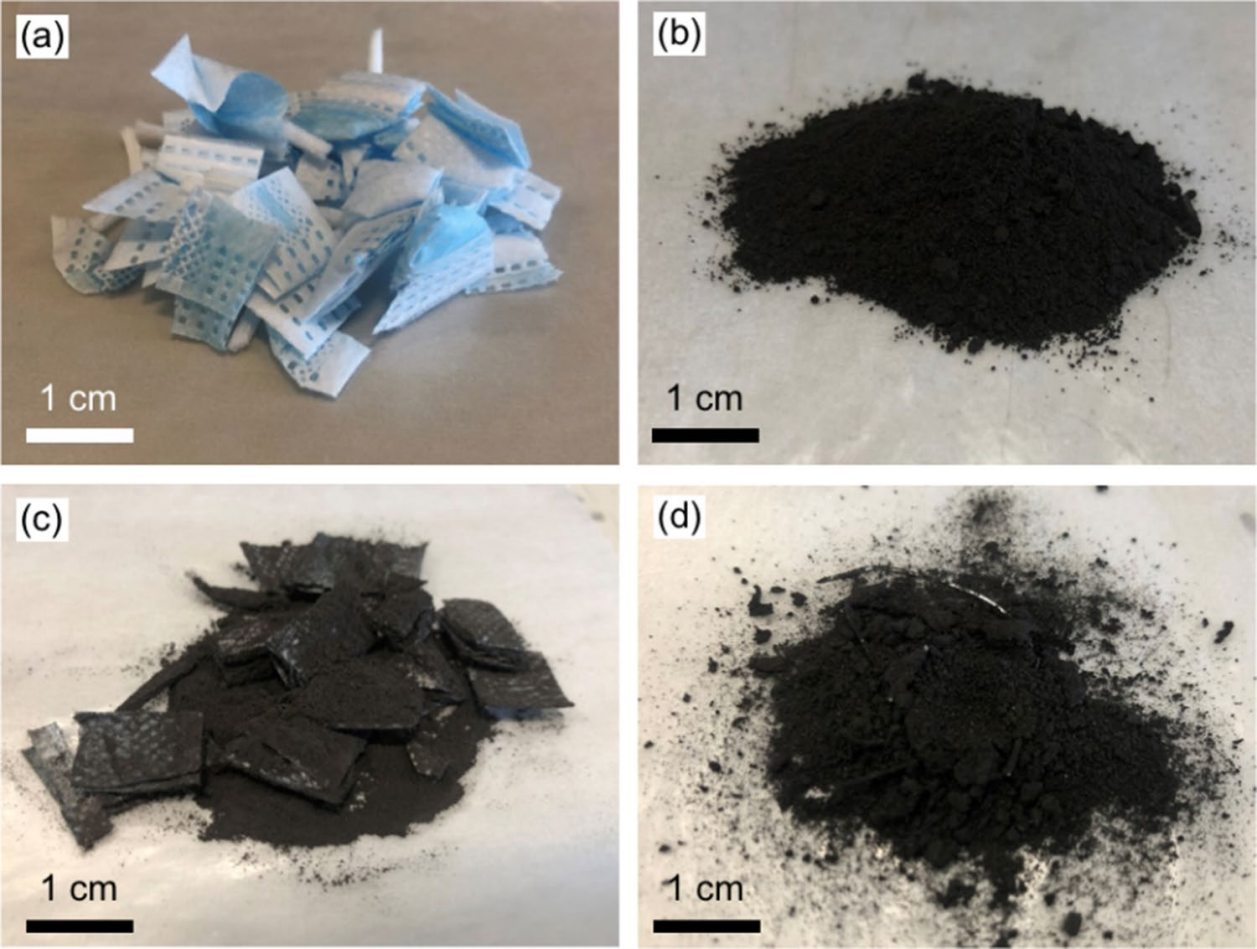
Materials at various stages of the milling process. (**a**) Coarsely cut untreated facemasks, (**b**) GCI coal, (**c**) cut facemasks and GCI coal before heat treatment and (**d**) facemasks and GCI coal after 1 h at 250 °C and before milling showing the morphology.

In the process, coarsely cut untreated facemasks (Fig. 2a) were added to GCI coal fines and charcoal (Fig. 2b) in a 20:80 weight ratio, and physically mixed (Fig. 2c). The resulting mixture was heat treated at 250 °C in a laboratory oven (in air) for one hour and then removed. While still hot, the mixture was stirred allowing the molten plastic to wet the carbon source and form a coarse powder (Fig. 2d). Once cooled to room temperature, the coarse powder was ball milled for 5 min at 500 rpm, whereby the powder fully passed a < 215 μm mesh, except for fragments of nose wire that remained in the mixture. These fragments may easily be recovered via screening or magnetic separation or can be left in place as an Fe source in a BF. The texture of the GCI coal/facemask sample after melt processing is that of a granular powder, which makes it suitable to be stored and transported, in particular via a conveyor belt, without creating an airborne powder hazard.

**Figure 3:**
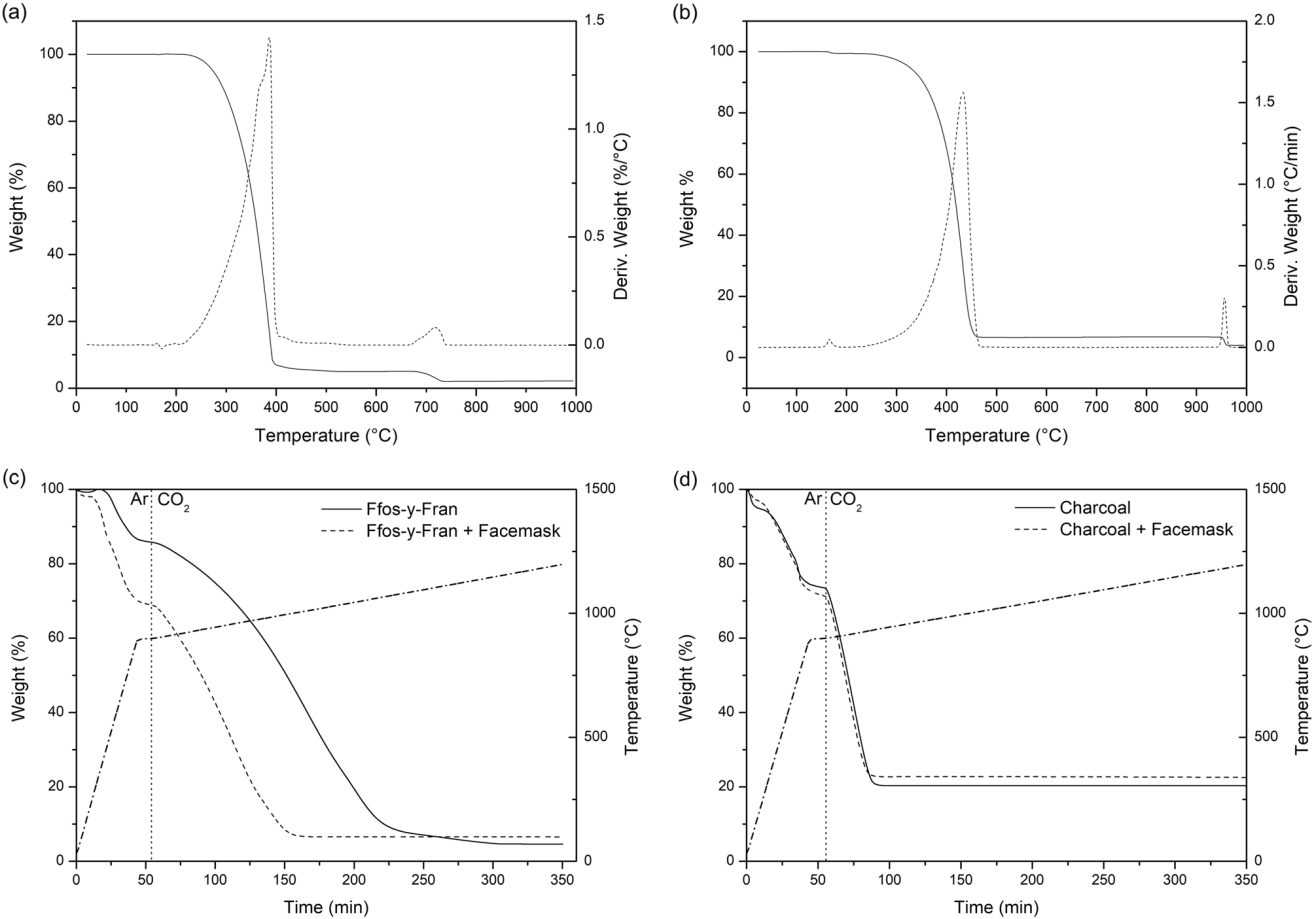
TGA-DTG curve for (**a**) untreated facemasks under a 100 cm^3^min^−1^ flow air, (**b**) untreated facemasks under a 100 cm^3^min^−1^ flow carbon dioxide. DTG signals are denoted as dashed lines. TGA measurements to 900 °C under Ar and to 1200 °C under CO_2_ atmospheres for (**c**) GCI coal (solid line) compared to GCI coal/20 wt.% facemask blend (dashed line) and (**d**) Charcoal (solid line) compared to charcoal/20 wt.% facemask blend (dashed line).

TGA-DTG shows melting of the facemask occurs at 167 °C in air (Fig. 3a) without significant decomposition. The lack of decomposition upon heating to 250 °C is confirmed by the FT-IR of the facemask after heating to this temperature (Figure S1), which shows no change. This indicates that the process of heating the facemask with GCI coal (or charcoal) simply involves the melting of the plastic with the carbon. An important observation is that the oxidative degradation mass loss of the facemask plastic begins at T > 250 °C, therefore this is the highest realistic temperature at which the milling procedure can be performed without substantial degradation of the mask material.

**Figure 4:**
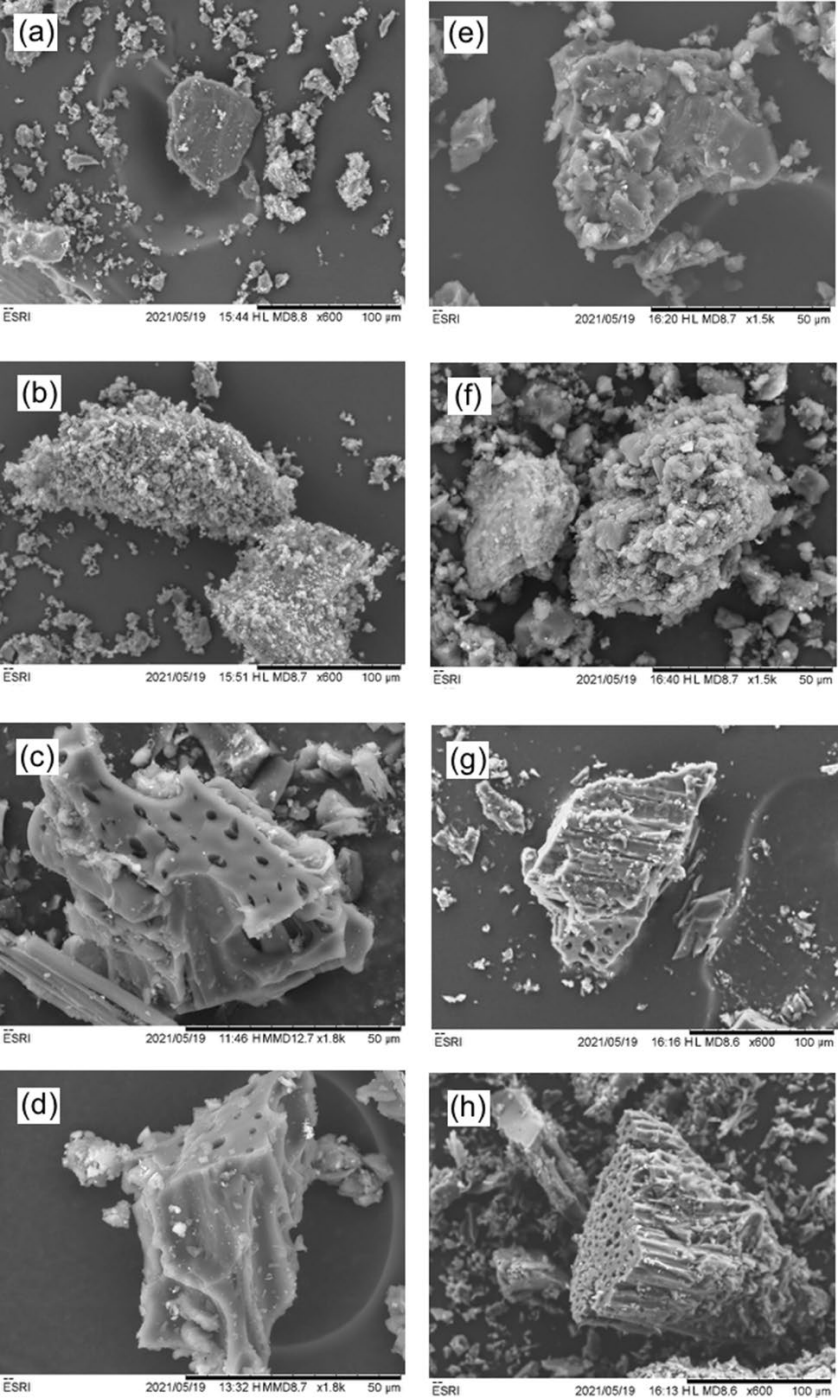
SEM images of samples of carbon and carbon/facemask melt blends. (**a**) GCI coal, (**b**) GCI coal with 20 wt.% facemask plastic, (**c**) charcoal, and (**d**) charcoal with 20 wt% added facemask plastic. (**e**) GCI coal after pyrolysis to 500 °C, (**f**) GCI coal with 20 wt.% facemask plastic after pyrolysis to 500 °C, (**g**) charcoal after pyrolysis to 500 °C, and (**h**) charcoal with 20 wt% added facemask plastic after pyrolysis to 500 °C.

The morphology of the resultant melt-blend materials was compared to that of the untreated carbon materials by SEM as shown in Fig. 4. The GCI coal samples (Fig. 4a) shows the flaky structure typical of raw coals with limited porosity^25^. The charcoal sample (Fig. 4c) appears significantly more porous as the material retains some of the cellular structure of the wood which it is derived from.

Comparing the untreated coal and charcoal to the samples treated with facemask plastic (Fig. 4b,d, respectively) it can be seen that the plastic readily wets and coats the coal particles while in the molten phase. Further to this, the mixing step disperses the molten polymer across the coal surface.

In order to determine the suitability of the coal/facemask melt blended product as an alternative reductant, TGA analysis has been performed under suitable conditions, in this case a CO_2_ atmosphere. Generally, CO_2_ gasification is the rate determining step in iron reduction within self-reducing agglomerates because it is so endothermic and kinetically slow. This is because the oxygen partial pressure is very low in the pellet bed of the RHF and gasification of carbon to carbon monoxide within the pellets by CO_2_ dominates over direct solid–solid reduction of metal oxides by fixed carbon.

TGA shows combustion of the facemask occurs at 382 °C in air (Fig. 3a); as may be expected this decomposition temperature is increased to 447 °C under inert atmosphere (Figure S2). In both cases a second smaller decomposition occurs at 700 °C leaving a residue of 3.06% (4.84% under Ar). As decomposition occurred under both oxidizing and inert conditions it is likely that this is a carbonate decomposition. Under the CO_2_ atmosphere (Fig. 3c) the second decomposition step observed did not occur until 955 °C suggesting that a high partial pressure of CO_2_ suppressed the decomposition step, supporting the observation that this is a carbonate decomposition. The ash content following oxidation at 1000 °C as determined in Fig. 3a was 2.20%, and by subtracting this value from the 4.84% mass remaining after heating under inert conditions to 1000 °C suggests that 2.64% of the total mass of the facemasks is available as fixed carbon (the solid, combustible, carbonaceous residue that remains after heating coal or organic material to high temperatures, after the pyrolysis of all volatile matter has taken place). The results suggest very little fixed carbon remains after the pyrolysis process for facemask plastic under inert conditions, in good agreement with pyrolysis tests performed by Jung et al. on the polypropylene layer of N95 masks^10^. Fixed carbon content is commonly used as a comparative metric in iron production, as it is effectively a measure of the solid carbon available for reduction of oxides at ironmaking reaction temperatures.

Analysis of the ash residue left from combustion under oxidizing conditions by SEM–EDX (Figure S3) indicates it to be primarily calcium(II) oxide (CaO) and calcium carbonate (CaCO_3_) which is consistent with the use of calcium carbonate as a filler to polypropylene and to enhance rheological properties during the manufacturing process^26,27^. This supports the observation that the mass loss at ~ 700 °C observed in Fig. 3 is decomposition of CaCO_3_. Calcium carbonate is commonly used within steelmaking as a supplementary flux^28^, so a CaO based ash from the facemask plastic would not present a major issue for steelmaking application from the standpoint of the introduction of far more undesirable elements in ash such as K, Na and Zn.

To replicate the oxidative behaviour of chars in the RHF the TGA of facemask blends was collected under an experimental program in which the initial rapid ramping period from ambient temperature to 900 °C occurs under inert atmosphere. After a stabilization period of 10 min at 900 °C, to allow for samples to equilibrate, the gas flow was switched to CO_2_ to initiate the gasification reaction and the samples were ramped to 1200 °C at various rates between 1–10 °Cmin^−1^. The TGA plots of GCI coal/20 wt.% facemask blend, in comparison with that of the untreated GCI coal are shown in Fig. 3c. The comparable plots for charcoal and charcoal/facemask blend are shown in Fig. 3d. The GCI coal samples shows pyrolysis of volatile organic compounds beginning at 382 °C with a mass loss of 13.8%, which is likely a complex mixture of volatile organic compounds present in the coal. However, for the facemask treated samples of GCI coal a far greater pyrolysis mass loss can be observed (29.1%) which is related to the simultaneous pyrolysis of the facemask plastic alongside the coal volatiles. Similarly, the facemask treated charcoal shows a greater mass loss compared to the untreated material at pyrolytic temperatures, but the difference is less stark (29.6% versus 20.6%). This observation suggests that the plastic has volatilized, and the products have departed from the reaction zone of the analyser long before CO_2_ oxidation is initiated.

It can be seen from Fig. 3c that the rate of CO_2_ oxidation of GCI coal was substantially increased by melt-blending with the facemask, with the untreated coal fully converting at 1155 °C compared to 993 °C for the facemask treated material. Interestingly, the treated charcoal performance is essentially unchanged compared to the untreated material, except for additional ash content. The samples completely reacted at 936 °C (untreated charcoal) and 917 °C (charcoal/facemask blend), respectively.

SEM images of the materials following pyrolysis at 500 °C are shown in Fig. 4. Little difference in the structure is observed between the charcoal (Fig. 4g) and facemask treated charcoal (Fig. 4h), both materials still exhibit the cellular structure and large pores characteristic of charcoal. In contrast, the pyrolyzed GCI coal sample treated with facemask plastic (Fig. 4f) appears significantly more amorphous than the GCI coal that was not treated (Fig. 4e). This change in surface morphology suggests that the action of plastic pyrolyzing from the surface of the coal during heat treatment is altering the surface structure of the coal. This textural change on the surface of the material may be responsible for the differences observed in CO_2_ reactivity.

Surface area analysis of the samples (Table 1) provides important insight into the mechanism of the increased reactivity observed in the GCI coal. Firstly, it is seen that pre-pyrolysis the samples mixed with facemasks have a dramatically increased surface area. We propose this is due to the adhesion of polymer particles to either the coal or charcoal particles creating a rough surface on the carbon particles. Following pyrolysis, however, the increased porosity observed in the pre-pyrolysis charcoal samples is eliminated and the charcoal and facemask treated charcoal exhibit essentially identical surface areas (Table 1). For the GCI coal however, it appears the mechanical and/or chemical action of the plastic pyrolysis has had a roughening effect on the relatively non-porous surface of the material leaving the post pyrolysis facemask treated sample significantly more porous than the sample without facemask plastic addition, in agreement with observations made using SEM in Fig. 4. A similar trend is seen in pore volume analysis performed using DFT, as the pyrolyzed GCI coal sample with 20 wt.% added facemask shows substantially higher pore volume than the untreated sample.

**Table 1:** Surface area (BET) and pore volume (DFT) analysis for pre- and post-pyrolysis of samples carbon and carbon/facemask samples.

Sample	Surface area (m^2^g^−1^)		Pore volume (cm^3^g^−1^)	
	Pre-pyrolysis	Post-pyrolysis^b^	Pre-pyrolysis	Post-pyrolysis^b^
GCI coal	5.498	6.265	0.036	0.032
GCI coal/facemask^a^	12.789	13.544	0.048	0.046
Charcoal	33.517	31.056	0.034	0.067
Charcoal/facemask^a^	75.238	31.067	0.055	0.071

^a^20 wt.% facemask added to the carbon source.

^b^500 °C in argon.

Pore development of coals as volatile compounds are lost from the material is a critical factor in gasification reactivity^29^, and thus surface area measurements can be useful for estimating reactivity. It is important to note, from the work of Sexton et al., that this relationship between surface area and CO_2_ activity was not found to be absolute and that the intrinsic chemical structure of the char material plays a substantial role in CO_2_ gasification reactivity. The chemical composition of the ash in the material also plays a key role: alkali and alkaline earth metals such as Na, K and Ca are extremely catalytically significant in CO_2_ gasification of coals^25,30^.

In order to gain further insight into the behaviour of the coal/facemask blend with regard to its potential application in RHF, the kinetics of the CO_2_ gasification step were investigated in detail. In this regard, CO_2_ gasification is an extremely complicated chemical process, many different chemical compounds in the complex starting material are reacting simultaneously and the mechanism of reaction likely changes with the degree of conversion; however, model-free kinetics allow for estimation of Arrhenius parameters without the assumption of a specific reaction mechanism and has previously been utilized to characterize the kinetics of pyrolysis of waste facemasks^31^.

Methods such as the Friedman method^32^, Flynn–Wall–Ozawa method (FWO)^33,34^, and the Kissinger Akahira Sunose method (KAS)^35^ are all commonly used iso-conversional methods of determining kinetic factors, primarily activation energy (E_a_). Boudouard reaction energy for CO_2_ gasification of carbon materials has been shown to vary substantially with conversion degree^36^. For this reason, the Friedman method was selected as the FWO and KAS methods are derived from systems where activation energy does not vary with reaction progress.

As changes in mechanism can be factored into the model free method used, CO_2_ is presumed to be in large excess, and the reverse Boudouard reaction is assumed to be negligible due to the high purge flow rate through the thermogravimetric analyser. As such the CO_2_ gasification of biomass/char/coal can be simplified to Eq. (1).

1 $${\text{C}}_{{{\text{fix}}}} \left( {\text{s}} \right) \, + {\text{ CO}}_{{2}} \left( {\text{g}} \right) \to {\text{2CO}}\left( {\text{g}} \right)$$

The general kinetic expression for decomposition gas–solid reactions is described in Eq. (2)^35^, where *t* is time, *k*(T) is the temperature dependent kinetic constant, f(α) is the expression of the reaction model, dα/dt is the rate of conversion with respect to time and α is the conversion degree as described in Eq. (3), where m_o_ is the samples initial mass before reaction initiation, m_t_ is the mass of the sample at time t and m_f_ is the final mass of the sample after the reaction has abated.

2 $${\text{d}}\alpha /{\text{d}}t = k\left( {\text{T}} \right){\text{f}}\left( \alpha \right)$$

3 $$\mathrm{\alpha }=\frac{{\mathrm{m}}_{0}-{\mathrm{m}}_{\mathrm{t}}}{{\mathrm{m}}_{0}-{\mathrm{m}}_{\mathrm{f}}}$$

By substituting the Arrhenius equation into Eq. (2), a general expression to calculate kinetic factors E_a_ and A is given in Eq. (4), where A is the preexponential factor (min^−1^), E_a_ is the activation energy of the conversion (kJmol^−1^) and T is absolute temperature (K).

4 $$\frac{\mathrm{d\alpha }}{\mathrm{dt}}=\mathrm{f}\left(\mathrm{\alpha }\right)\mathrm{Aexp}\left(-\frac{{\mathrm{E}}_{\mathrm{a}}}{\mathrm{RT}}\right)$$

The expression as proposed by Friedman involves taking the natural logarithm of Eq. (4) to yield Eq. (5)^32^^,^^37,38^.

5 $$\mathrm{ln}\left(\frac{\mathrm{d\alpha }}{\mathrm{dt}}\right)=\mathrm{ln}\left[\mathrm{Af}\left(\mathrm{\alpha }\right)\right]-\frac{{\mathrm{E}}_{\mathrm{a}}}{\mathrm{RT}}$$

**Figure 5:**
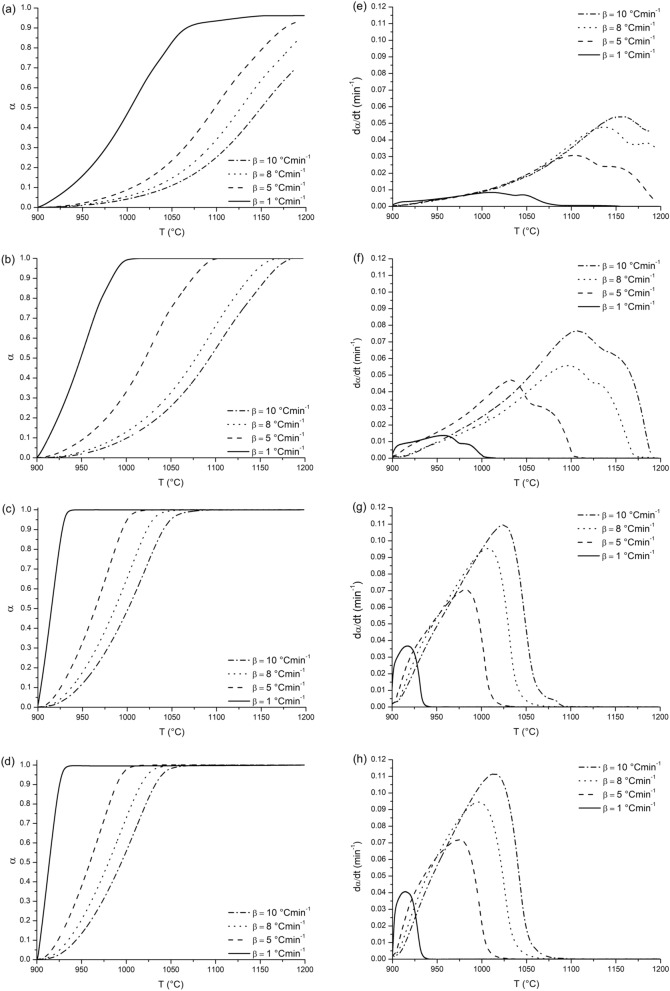
Plots of α and dα/dt against T (°C) for different heating rates. (**a**) GCI coal, α against T. (**b**) GCI coal melt-blended with 20 wt.% facemask material, α against T. (**c**) Charcoal, α against T. (**d**) Charcoal melt-blended with 20 wt.% facemask material, α against T. (**e**) GCI coal, dα/dt against T. (**f**) GCI coal melt-blended with 20 wt.% facemask material, dα/dt against T. (**g**) Charcoal, dα/dt against T. (**h**) Charcoal melt-blended with 20 wt.% facemask material, dα/dt against T.

The values of E_a_ with respect to conversion degree α can be determined the plot of ln(dα/dt) against 1000/T at a constant conversion value for several experiments with varying heating rate β (°Cmin^−1^), where the slope of each iso-conversional line corresponds to − E_a_/R. A typical plot of α versus T for GCI coal and the GCI coal/facemask melt blend is shown in Fig. 5a,b, respectively, while the analogous plots for the charcoal samples are given in Fig. 5c,d. Charcoal is seen to be significantly more reactive to CO_2_ than GCI coal, this is likely related to the material’s intrinsic high degree of porosity as observed in BET and SEM analyses (Table 1., Fig. 4).

By plotting dα/dt against T for the samples, the difference in maximum rates between the samples is clearer and is shown in Fig. 5e–h. The gasification of charcoal (Fig. 5g) appears to take place in a single stage whereas the twin peaks observed in the GCI coal (Fig. 5e) and GCI coal with 20 wt% added facemask (Fig. 5f) suggests a more complicated multi-step mechanism of CO_2_ gasification.

**Figure 6:**
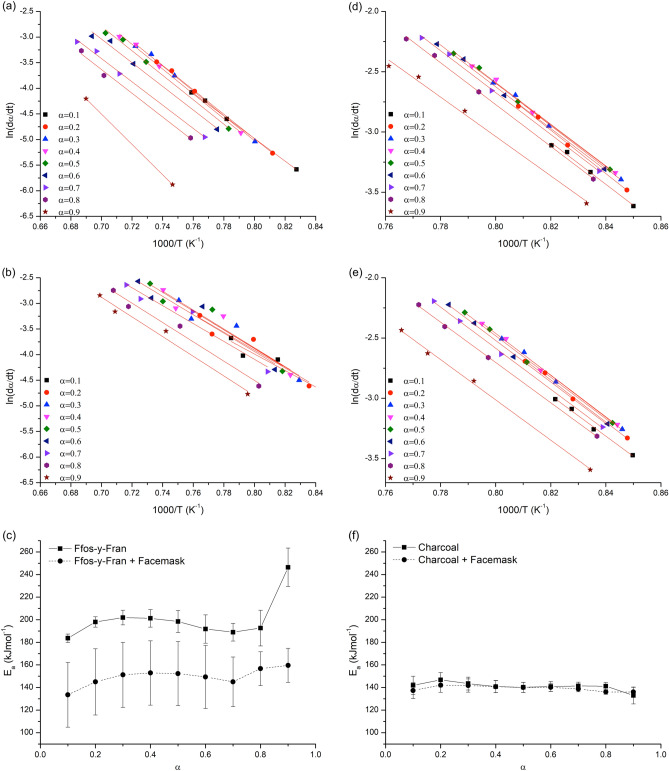
Friedman plots and calculated activation energy (E_a_) against α for the combustion of carbon and carbon/facemask melt blends in CO_2_ atmosphere. (**a**) Friedman plot for GCI coal. (**b**) Friedman plot for GCI coal/facemask melt blend. (**c**) Activation energy against α for GCI coal and GCI coal/facemask melt blend. (**d**) Friedman plot for charcoal. (**e**) Friedman plot for charcoal/facemask melt blend. (**f**) Activation energy against α for charcoal and charcoal/facemask melt blend.

Charcoal and facemask treated charcoal show extreme similarity in terms of CO_2_ reactivity, with the DTG signals appearing nearly identical. This is in stark contrast to the GCI coal samples where maximal rate and reaction completion temperature are significantly improved (lowered) by addition of facemask plastic. Friedman plots of ln(dα/dt) against 1000/T at iso-conversions of α = 0.1–0.9 for each sample are shown in Fig. 6 and calculated activation energies for each of the samples based on the iso-conversional line slopes in Fig. 6c,f.

Activation energies for CO_2_ gasification of charcoal ranged from 133 to 147 kJmol^−1^ compared with 136 to 142 kJmol^−1^ for facemask treated charcoal in reasonable agreement with Dai et al.’s calculated CO_2_ gasification activation energies for various biomass chars^39^. The tight range of activation energies at different stages of conversion suggests a consistent gasification mechanism for both treated and untreated charcoal. The results imply that the treatment with facemask plastic for charcoal has little to no effect on CO_2_ gasification reactivity.

Activation energies for GCI coal ranged from 184 to 246 kJmol^−1^, with an apparent shift in the reaction mechanism at higher levels of conversion, where a sharp increase in E_a_ is observed for α = 0.9. These results are broadly similar to calculated activated energies for Indian coals^40^ and coal chars^37^ but the range of reported values for the Boudouard reaction is extremely wide in literature, due to its dependence on structural factors in the material. GCI coal treated with facemask plastic showed a marked decrease in activation energy across the entire conversion range when compared with the untreated material, with values spanning 134–160 kJmol^−1^.

That the addition of facemask plastic to coal can increase its reactivity so decidedly, even though the plastic has long since decomposed and volatilized before the reaction gas was introduced to the thermogravimetric analyser implies that either, the ash residue of the facemasks is acting in a catalytic manner to accelerate CO_2_ gasification or that the action of the plastic in the material vaporizing and diffusing away from the solid is introducing some form of chemical or structural change in the material that is leading to increased CO_2_ gasification reactivity (Fig. 6c,f).

## Discussion

The observation that charcoal is not seen to increase in reactivity with the addition of facemask plastic, but the GCI coal sample does, has two potential explanations. The first is that the far higher initial porosity of the facemask treated charcoal sample led to the facemask treatment doing little to increase porosity further, and therefore have no tangible effect on CO_2_ gasification reactivity. Secondly, if the deposition of catalytic CaO ash following pyrolysis of the facemask plastic is the dominant mechanism of increased CO_2_ reactivity, this may be masked due to the high ash content of the charcoal (18.7%) having such a significant catalytic effect on CO_2_ reactivity that the further small addition of facemask ash has no tangible effect.

It is likely there is a combination of catalytic and pore development factors responsible for the observed increase in reactivity of facemask treated GCI coal. Nevertheless, the CO_2_ gasification reactivity of a sample of a low volatile content coal was dramatically improved by the addition of waste facemask plastic, as determined through non-isothermal thermogravimetric measurements. This was not observed in a substantially more porous, higher volatile content and higher ash content charcoal material. The results suggest a potentially synergistic mechanism where the deposition of CaO based ash following the pyrolysis of the facemask plastic, as well as a physical change in the structure of the coal causing increased porosity both play a role in increasing the reactivity of the material in the CO_2_ gasification reaction^41^. Further study is required to fully parse these two competing mechanistic explanations.

**Figure 7:**
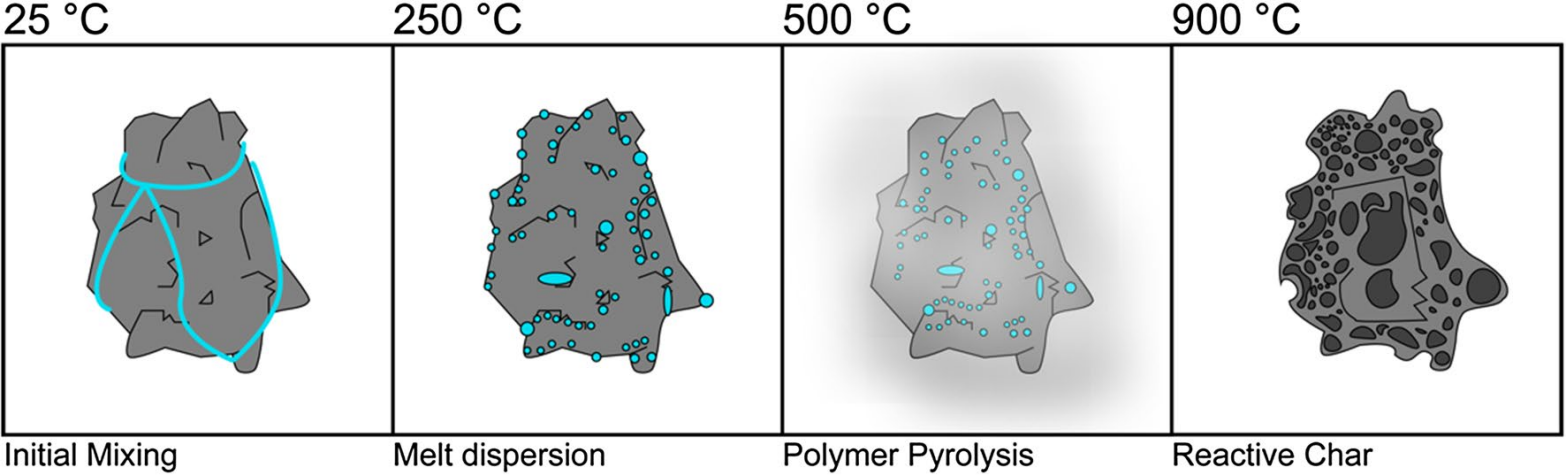
Proposed process schematic showing the effect of the polymer fabric of facemasks on a coal particle as it undergoes heat treatment.

This increase in CO_2_ gasification reactivity in a comparatively low volatile content coal is significant for the purposes of iron reduction in a RHF, where the gasification reactivity of the carbon is seen to dominate Fe reduction kinetics during much of the reduction reaction^18^. This is especially significant because the carbon and hydrogen content of the facemask plastic is also available for reduction of iron oxides, meaning not only that a small amount of required coal is displaced, but also that the utilization of the coal still required is more efficient. Steer et al. described a close relationship between increasing volatile matter content of injection coals and reduced burnout time (and therefore improved combustion reactivity) using a drop tube furnace (DTF) to emulate the conditions of the raceway of a BF and attributed this increase to increasing particle surface area and additional heat supplied by combustion of coal volatiles^41^. We propose that the action of dispersing molten facemask plastic across the surface of low volatile content coal is acting in a manner similar to intrinsic volatile matter within the coals, opening pores in the material and increasing the reactive surface area of the particles. An overall schematic for the process is shown in Fig. 7.

The results are favourable for the purposes of increasing CO_2_ reactivity of low-quality carbonaceous materials for RHF processing of steelmaking by-product sludges. Increased char reactivity may lead to improved iron and zinc reduction rates in self-reducing agglomerates and the addition reducing gases provided by the facemask plastic would also contribute additional reductant; however, through exploring the properties of the facemask treated GCI coal for RHF application it became apparent that the material may be a good candidate for direct injection at the BF tuyere as well.

The injection of coal through the tuyeres into the blast furnace has become extremely commonplace in the global steel industry as a means of reducing demand for expensive and environmentally costly coke in iron production^42,43^. BF coal injection rates have been steadily increasing for decades, from 60–80 kgtHM^−1^ in the 1980s to over 200 kgtHM^−1^ in some plants^44^. An extremely important consideration when selecting candidate coals for injection to the BF is the reactivity of the char produced following injection. Coal that enters the furnace via a tuyere experiences oxidizing conditions and an extremely high heating rate as the material enters the raceway. Any coal particles that are not completely reacted at this stage leave the raceway region as a partially burnt-out char where gasification under the prevailing gas condition of the furnace becomes dominant.

In order to achieve the maximum possible injection rate, it is critical that the injected material reacts and gasifies in as short a time period as possible^45^, de Lourdes Ilha Gomez et al. stipulated that “The more reactive a char is, the better is the effect in the replacement of coke” with respect to pulverised coal injection and the BF^46^. Char that is not completely consumed during PCI is obviously economically unfavourable as it is underutilized for the purposes of iron reduction, but much more significantly, unburnt material can accumulate within the furnace burden. This can have substantial negative effects on burden permeability, furnace stability, temperature distribution, coke erosion and char carryover^47^. Injection rates to a BF can be limited by a number of other factors such as S and ash input but one of the key reactivity criteria that has an established effect on burden permeability is the insufficient gasification of chars formed after injection to the furnace^29^. Therefore, the improved gasification reactivity exhibited by the facemask treated GCI coal may have potential application in improving coal reactivity during BF injection and therefore present a method of making cheaper, less reactive coals more attractive for injection or potentially increasing the maximum injection rate without negative effects on furnace operation.

Globally, plastic injection systems are also emerging capable of injecting plastic to the furnace, reducing fossil fuel requirements and sparing plastic waste from landfill or entering the ocean^48^. These modified plants are capital intensive, however, requiring specialist support plant and tuyere modifications to be capable of handling the extreme heterogeneity of a recycled plastic feed^48^, subject to further trials the method of melt-blending described herein may present a way of introducing plastics to a BF with minimal plant modification. As waste facemask plastic is unfortunately, readily available in the wake of the pandemic of SARS-CoV-2 and its variants, its application in the iron and steelmaking industry is a positive avenue of disposal to prevent material being introduced into rivers and oceans while displacing environmentally damaging fossil fuels from the steelmaking process.

Further study into applications of the material is required to assess whether the improved CO_2_ gasification reactivity observed in this thermogravimetric study would indeed translate into improved reduction performance in an RHF. Drop tube furnace trials designed to replicate the unique chemical conditions of the BF tuyere will elucidate whether the improved char reactivity exhibited in this study may translate into the BF environment and make the material a suitable auxiliary BF injectant.

## Methods

Disposable, non-medical grade face coverings were used for the purpose of this study. Masks produced by the Changzhou Huangshi Packaging Printing Co. Ltd. were coarsely chopped into squares using scissors without any prior disassembly. Charcoal was purchased from Fisher Scientific, and the coal used was a typical BF injection coal. Both materials were dried at 90 °C for 12 h and pulverized and sieved to < 215 μm. Proximate analysis of the GCI coal and charcoal samples^49^, along with the carbon and sulphur analysis results are provided in the Supplementary Materials.

Thermogravimetric experiments were carried out using a TA Instruments SDT Q600 analyser. Experiments were performed on 20 mg ± 0.5 mg of sample, using alumina sample crucibles. For non-isothermal tests, samples were ramped to reaction temperature (900 °C) under protective cover of argon (100 cm^3^ min^−1^) at a rate of 20 °C min^−1^ and held isothermally for a period of 10 min at reaction temperature. The purge gas was then changed to CO_2_ at a rate of 120 cm^3^ min^−1^ to initiate the reaction before ramping the analysis temperature from 900 °C to 1200 °C at a range of heating rates (1, 5, 8 and 10 °C min^−1^)^50^. Surface area analysis was performed using a Quantachrome Nova 2000e analyzer using N_2_ as an adsorbate gas. Around 130 mg of sample was degassed under vacuum at 130 °C overnight. BET surface area was calculated in the 0.09237–0.24399 P/P_0_ range for each of the samples. FTIR measurements were taken using a Thermofisher Scientific Nicolet iS10. Scanning electron microscopy was performed on a Hitachi TM3030. Carbon and sulphur were determined via combustion analysis using an ELTRA CS500 C/S Analyzer.

Ball milling of samples was performed in a Fritsch Pulverisite 6 ball mill at 500 rpm. Bulk pyrolysis experiments were performed using a carbolite MTF 12/38/400 controlled atmosphere tube furnace heated to 500 °C at a rate of 5 °C min^−1^ under the protection of 1 dm^3^ min^−1^ pureshield argon to heat a pellet of material (16 mm diameter, 2.5 g, 975 bar, 60 s residence time) produced using a Retsch PP 25 Pellet Press. Samples were cooled in the furnace to ambient temperature for pulverizing and further analysis.

## Supplementary Information

Supplementary Information 1.

Supplementary Information 2.

## Data Availability

All data are available in the main text or the supplementary materials.
