# Supplementary material for: Facemasks and ferrous metallurgy: improving gasification reactivity of low-volatile coals using waste COVID-19 facemasks for ironmaking application
Source: Sci Rep. 2022 Feb 17;12:2693. doi: 10.1038/s41598-022-06691-w (PMC8854564; doi:10.1038/s41598-022-06691-w)
Supplement: Supplementary file 2 — Supplementary Information 2. [file 41598_2022_6691_MOESM2_ESM.docx]

Facemasks and ferrous metallurgy: improving gasification reactivity of low-volatile coals using waste COVID-19 facemasks for ironmaking application

Daniel J. C. Stewart,^1^ Lucy V. Fisher,^1^ Michael E. A. Warwick,^1^ David Thomson,^2^ and Andrew R. Barron^1,3,4,5^*

^1^Energy Safety Research Institute, Swansea University Bay Campus, Swansea, SA1 8EN, UK. ^2^Tata Steel Strip Products UK, Port Talbot, SA13 2NG, UK, ^3^Arizona Institutes for Resilience (AIR), University of Arizona, Tucson, Arizona 85721, USA, ^4^Department of Chemistry and Department of Materials Science and Nanoengineering, Rice University, Houston, Texas 77005, USA, ^5^Faculty of Engineering, Universiti Teknologi Brunei, Brunei Darussalam

Correspondence and requests for materials should be addressed to A.R.B. ([a.r.barron@swansea.ac.uk](mailto:a.r.barron@swansea.ac.uk) or [andrewbarron@arizona.edu](mailto:andrewbarron@arizona.edu))





Figure S1. FTIR spectra for untreated facemasks (top) and facemasks that have been held at 250 °C for 1 hour (bottom).





Figure S2. TGA-DTG curve for untreated facemasks under a 100 cm^3^/min flow argon.


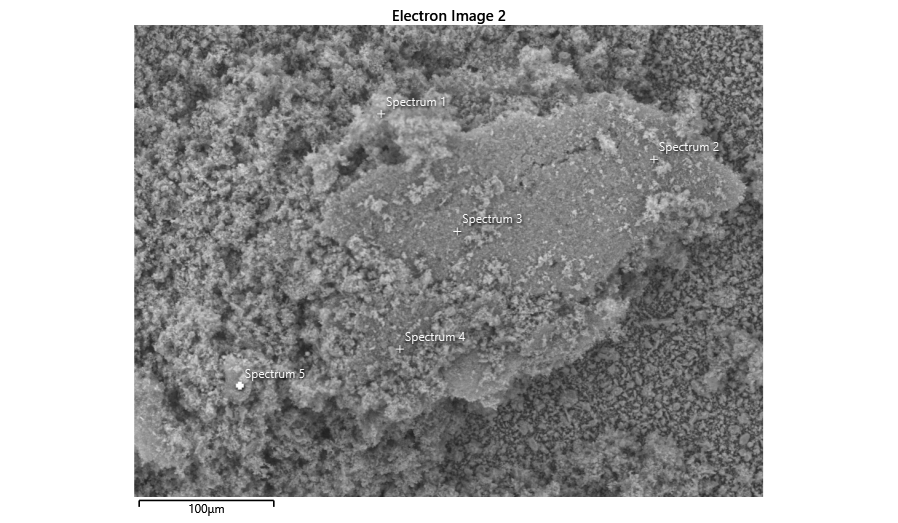


Figure S3. SEM of ash produced by combustion of untreated facemasks.

Table S1. Proximate Analysis of the coal and charcoal samples

| Material | Moisture (wt%) | Volatile Matter (wt%) | Fixed Carbon (wt%) | Ash (wt%) |
| --- | --- | --- | --- | --- |
| Ffos-y-Fran | 0.67 | 12.71 | 78.75 | 7.77 |
| Charcoal | 3.91 | 23.71 | 53.46 | 18.64 |

Table S2. Carbon and sulphur analysis of charcoal, Ffos-y-Fran coal and coarse facemasks.

| Material | Carbon (wt%) | Sulphur (wt%) |
| --- | --- | --- |
| Ffos-y-Fran | 90.08 | 1.308 |
| Charcoal | 63.69 | <0.01 |
| Facemasks | 85.68 | <0.01 |

Table S3. EDX analysis of ash produced by combustion of untreated facemasks.

| Element | Composition(wt.%) | σ (wt.%) |
| --- | --- | --- |
| Al | 0.34 | 0.04 |
| C | 9.64 | 1.20 |
| Ca | 50.24 | 9.49 |
| Cu | 0.01 | 0.02 |
| Mg | 0.10 | 0.09 |
| O | 38.52 | 8.50 |
| Si | 0.17 | 0.07 |
| Ti | 0.79 | 0.49 |
| Zn | 0.08 | 0.18 |

Data S1.xlx (Separate file)

The datafile containing all thermogravimetric data, Friedman plots, calculations etc. is supplied in excel format.
